# BoDV-1 Infection Is Associated with Altered Prion Protein Glycoform Maturation and Reduced ETS1-MGAT5/GnTV Pathway Activity in Rat C6 Astroglial Cells

**DOI:** 10.3390/microorganisms14081759

**Published:** 2026-08-10

**Authors:** Akikazu Sakudo, Kazuyoshi Ikuta

**Affiliations:** 1 School of Veterinary Medicine, Okayama University of Science, Imabari 794-8555, Ehime, Japan; 2 Research Institute for Microbial Diseases, Osaka University, Yamada-oka, Suita 565-0871, Osaka, Japan

**Keywords:** Borna disease virus, BoDV-1, glycosylation, prion, PrP

## Abstract

Borna disease virus 1 (BoDV-1) is a noncytolytic, neurotrophic virus that causes neurological disorders, where astrocytes contribute to neuropathology. Cellular prion protein (PrP^C^) is involved in pro-survival signaling and antioxidative defense. Using rat astroglial C6 cells as an *in vitro* model, we investigated whether persistent BoDV-1 infection alters *N*-glycan maturation of PrP^C^. Western blotting showed that PrP^C^ shifted toward lower-molecular-weight glycoforms as cell density increased. This tendency was more evident in BoDV-1-infected C6 cells without detectable changes in total surface PrP^C^ expression. Two-dimensional polyacrylamide gel electrophoresis showed a shift of PrP^C^ toward lower molecular weight and more basic isoelectric points. Conventional RT-PCR detected reduced signals of MGAT5, which encodes *N*-acetylglucosaminyltransferase V (GnTV), whereas MGAT1–4 signals were not markedly altered. In addition, GnTV overexpression induced a higher-molecular-weight shift of PrP^C^. PHA-L4 precipitation followed by anti-PrP immunoblotting showed reduced β1-6-branched complex *N*-glycan signals associated with PrP^C^. BoDV-1 infection also decreased the RT-PCR signal of E26 transformation-specific 1 (ETS1) as well as reduced ETS-dependent promoter activity and superoxide dismutase activity. These findings suggest that persistent BoDV-1 infection may impair PrP^C^ *N*-glycan maturation through suppression of the ETS1–MGAT5/GnTV pathway activity, thereby potentially affecting astroglial antioxidative capacity.

## 1. Introduction

Prion diseases, also referred to as transmissible spongiform encephalopathies, are fatal neurodegenerative disorders characterized by the conformational conversion of the host-encoded cellular prion protein (PrP^C^) into an abnormal isoform termed PrP^Sc^ [1,2,3]. This conversion is associated with a marked structural transition from an α-helix-rich PrP^C^ conformation to a β-sheet-rich PrP^Sc^ conformation, as shown by biophysical and structural analyses of prion proteins (PrPs) [4,5]. Although PrP^C^ and PrP^Sc^ share the same amino acid sequence, their distinct conformational states and assembly properties underlie the infectious propagation and neurotoxicity attributed to PrP^Sc^ [1,2,6]. PrP^C^ is a highly conserved glycosylphosphatidylinositol (GPI)-anchored glycoprotein expressed on the surface of neurons and glial cells [7,8,9]. Several lines of evidence suggest PrP^C^ is involved in neurite outgrowth, cell–cell adhesion, synaptic regulation, immune cell activation, secondary lymphoid tissue formation, metal homeostasis, maintenance of myelin integrity, and protection against oxidative stress [7,8,9,10,11,12,13,14].

A central biochemical feature of PrP^C^ is its heterogeneous *N*-linked glycosylation. The C-terminal domain of PrP^C^ contains two conserved *N*-glycosylation sites, generating di-, mono-, and unglycosylated PrP^C^ isoforms. A substantial body of evidence indicates that *N*-glycosylation of PrP is functionally important in prion biology. Glycoform ratios differ across brain regions, cell types, and lymphoid tissues and are frequently altered in prion-infected brains [15]. Moreover, prion strains can often be distinguished by characteristic PrP^Sc^ glycoform patterns following proteinase K digestion, reflecting differences in the relative abundance of the di-, mono-, and unglycosylated PrP^Sc^ molecular species [16]. Experimental studies further demonstrate that glycosylation influences cross-species conversion and host range [17]. Moreover, the stoichiometry of host PrP^C^ glycoforms modulates the efficiency of PrP^Sc^ formation *in vitro* [18]. In addition, the sialylation status of PrP *N*-glycans has been reported to affect prion replication kinetics and glycoform ratios [19]. Collectively, these findings indicate that *N*-glycosylation of PrP affects the conversion dynamics and strain-associated biochemical phenotypes rather than simply serving as a passive post-translational modification.

Beyond its relevance to conversion kinetics, glycosylation of PrP is linked to cellular homeostasis and stress susceptibility. PrP glycosylation is influenced by cellular processing conditions, which can change PrP conformation, trafficking, and neurotoxicity [20,21]. PrP^C^ has also been implicated in antioxidative defense through both intrinsic and ligand-mediated mechanisms. Binding of the co-chaperone stress-inducible phosphoprotein 1 (STI1) to PrP^C^ activates pro-survival signaling cascades and enhances resistance to oxidative injury [22,23,24]. STI1-PrP^C^ signaling has been further linked to increased cellular antioxidative capacity, in part through modulation of superoxide dismutase (SOD) activity [25] and suppression of reactive oxygen species accumulation [26]. Indeed, PrP expression has been shown to enhance SOD activity and PrP-deficient cells are more vulnerable to oxidative stress [26,27]. These findings raise the possibility that perturbations in PrP *N*-glycan maturation may compromise PrP-dependent cytoprotective pathways.

Complex *N*-glycan branching is a major determinant of glycoprotein function at the cell surface and is dynamically regulated by transcriptional and metabolic cues. *N*-acetylglucosaminyltransferase V (GnTV/MGAT5) catalyzes the formation of β1-6-branched complex *N*-glycans and thereby modulates receptor clustering, membrane retention, and signal transduction [28,29,30,31]. Notably, MGAT5 expression is transcriptionally regulated by transcription factors, and E26 transformation-specific 1 (Ets-1) has been shown to positively regulate MGAT5 promoter activity and mRNA expression [29]. MGAT5-dependent branching has been extensively studied in cancer and immune regulation [28,29,30,31]. More recently, however, MGAT5-dependent branching has also been found to be relevant to neural physiology. For example, MGAT5-mediated *N*-glycosylation is implicated in neural stem/progenitor differentiation and neurodevelopment [32]. These lines of evidence support the premise that perturbations of the ETS1-MGAT5 axis may selectively remodel β1-6 branching in neural cells under conditions of altered growth state or chronic stress.

Borna disease virus 1 (BoDV-1) is a non-cytolytic, highly neurotropic negative-strand RNA virus that establishes persistent infection in neurons and glial cells of the central nervous system [33,34]. Experimental models have shown that BoDV-1 infection can induce behavioral and neurological abnormalities and that astrocytes play critical roles in BoDV-1-associated neuropathology. For instance, transgenic expression of BoDV-1 phosphoprotein (P) in astrocytes causes pronounced neurological and behavioral phenotypes [35], and BoDV-1 P expression perturbs astrocytic growth factor-related signaling pathways [36]. More recently, transcriptomic analyses demonstrated broad and persistent changes in host gene expression induced by BoDV-1 infection in differentiated neuronal cells, with partial reversibility upon antiviral intervention [37]. In addition, bornaviruses have been reported to associate with neuronal DNA double-strand breaks during replication, providing a mechanistic basis for persistent host regulatory perturbation [38]. Together, these studies establish BoDV-1 as a potent and persistent modulator of neural and glial homeostasis.

Cell density was included as an experimental variable because PrP glycoform expression has previously been reported to vary with cell density and neuronal differentiation [39]. Moreover, formation of a confluent monolayer can remodel complex *N*-glycan branching of cellular glycoproteins including Na,K-ATPase β_1_ subunit and E-cadherin through changes in the expression of *N*-glycan-processing enzymes such as GnTV [40]. These observations suggest that the cellular growth state may influence PrP glycoform maturation and modify the effects of persistent BoDV-1 infection.

However, the molecular mechanisms by which BoDV-1 persistence and cellular growth state remodel *N*-glycans of PrP remain unclear. β1-6 branching of complex *N*-glycans is principally controlled by GnTV and its transcriptional regulator Ets-1 [29]. Moreover, it has been proposed that BoDV-1 can reprogram host transcriptional networks [37,41,42]. Thus, we hypothesized that BoDV-1 suppresses the ETS1-MGAT5 pathway, thereby impairing β1-6 branching and promoting accumulation of under-glycosylated PrP. We further postulated that such glycan remodeling could be linked to altered oxidative-stress phenotypes, consistent with the established involvement of PrP in cellular homeostasis [20].

In the present study, we examined how BoDV-1 infection and cell density regulate *N*-glycan maturation of PrP in rat astroglial C6 cells and have identified the glycosyltransferase pathway underlying these changes. C6 cells were selected because they provide a well-established and experimentally reproducible astroglial model of persistent BoDV-1 infection [43], allowing long-term comparisons between infected and uninfected cells under matched culture conditions. Using BoDV-1-infected C6 cells, we analyzed glycoform profiles of PrP by Western blotting and two-dimensional polyacrylamide gel electrophoresis (2D-PAGE), assessed PrP-associated β1-6 branched *N*-glycan signals by phytohemagglutinin L4 (PHA-L4) lectin precipitation followed by PrP immunoblotting, and investigated the expression and functional contribution of GnTV and Ets-1 using gain-of-function approaches as well as employing Ets-dependent promoter activity assays. This work aims to clarify how persistent neurotropic viral infection can reprogram astroglial glycosylation, with potential implications for both viral neuropathogenesis and prion biology.

## 2. Materials and Methods

### 2.1. Cell Cultures

Rat astroglial C6 cells (Database Name: Cellosaurus; Accession Number: CVCL_0194) [44], the transfectants, and the BoDV-1 (huP2Br strain [45])-infected C6 cells (C6/BoDV-1) were maintained in Dulbecco’s modified Eagle’s medium (DMEM) (D6046; Sigma-Aldrich, St. Louis, MO, USA) supplemented with 10% fetal calf serum (FCS) at 37 °C in a humidified 5% CO_2_ incubator. Persistently infected C6 cells were established after infection with the huP2Br [45] strain of BoDV-1 and passaged for more than 40 days.

### 2.2. Antibodies

For detection of GnTV, a rabbit anti-GnTV antibody (24D11; Fujirebio, Tokyo, Japan) was used. For BoDV-1 detection, anti-BoDV-1 p40 antibody [46] was used. SAF32 (SPI bio, Montigny le Bretonneux, France), recognizing the N-terminal region of PrP, and SAF83 antibody (SPI bio), recognizing the C-terminal region of PrP, were used. For internal control, anti-α-tubulin antibody (B-5-1-2; Sigma-Aldrich) was used. Horseradish peroxidase (HRP)-conjugated anti-mouse or anti-rabbit IgG (H+L) antibodies (Jackson ImmunoResearch, West Grove, PA, USA) were used for chemiluminescent detection.

### 2.3. Western Blotting

The procedures for extracting protein from cells using radio-immunoprecipitation assay (RIPA) buffer and subsequent sodium dodecyl sulphate polyacrylamide gel electrophoresis (SDS-PAGE) and immunoblotting with antibodies described above have been reported previously [47]. Briefly, cells were lysed in RIPA buffer containing 50 mM Tris-HCl (pH 7.4), 150 mM NaCl, 1% NP-40, 0.5% sodium deoxycholate, and 0.1% SDS together with a protease inhibitor cocktail (cOmplete EDTA-free protease inhibitor cocktail tablets; Roche, Basel, Switzerland). Protein concentration was determined using a Lowry assay (Bio-Rad DC protein assay kit; Bio-Rad, Hercules, CA, USA). Proteins (20 µg per lane) were separated by SDS-PAGE (8–12% acrylamide gradient gel) and transferred to a polyvinylidene difluoride (PVDF) membrane. The membrane was subsequently blocked overnight at 4 °C using 5% skimmed milk in phosphate-buffered saline (PBS) containing 0.05% Tween20. Blots were then incubated with either anti-GnTV antibody 24D11, anti-α-tubulin antibody, anti-BoDV-1 p40 antibody, anti-PrP antibody SAF32, or anti-PrP antibody SAF83 for 1 h at 37 °C. After washing three times with PBS containing 0.05% Tween20, the membranes were incubated with HRP-conjugated anti-mouse or anti-rabbit IgG (H+L) antibodies (Jackson ImmunoResearch) for 1 h at 37 °C. Signals were visualized by Enhanced Chemi-Luminescence (ECL) (GE Healthcare Technologies Inc., Chicago, IL, USA).

### 2.4. Densitometric Analysis of Blots

Blot images were analyzed using ImageJ software version 1.49v (National Institutes of Health, Bethesda, MD, USA). A rectangular selection of consistent width and height was applied to each lane, encompassing the full migration range of bands. Intensity profiles along the migration axis were then obtained using the Plot Profile function. The *x*-axis represents the electrophoretic position within the selected analysis range, expressed in pixels, where Pixel 0 was defined as a position on the lower-molecular-weight side of the selected range. For background correction, the minimum intensity value observed anywhere within the entire profile of each lane was identified and subtracted from every intensity value in the corresponding lane. The resulting profiles represent relative band intensity at each electrophoretic position (pixels).

### 2.5. 2D-PAGE

A confluent monolayer of C6 and C6/BoDV-1 cells on a 100-mm dish was harvested using a scraper. Equal numbers of cells (2.5 × 10^6^ cells per sample) were suspended in extraction buffer (8 M urea, 4% 3-[(3-cholamidopropyl)dimethylammonio]-1-propanesulfonate (CHAPS), 50 mM dithiothreitol (DTT)), sonicated, and centrifuged at 15,000 rpm for 5 min. The entire supernatant prepared from 2.5 × 10^6^ cells was used as the sample. Samples were mixed with rehydration buffer (8 M urea, 4% CHAPS, 50 mM DTT, 0.2% (*w*/*w*) ampholine (pH 3–10), 0.001% bromophenol blue) and then subjected to 2D-PAGE. Isoelectric focusing was carried out on pH 3–10 IPG strips (Bio-Rad) in the first dimension, followed by SDS-PAGE in the second dimension. Proteins were visualized by Coomassie Brilliant Blue R-250 staining or transferred for immunoblotting using anti-PrP antibody SAF32 or SAF83.

### 2.6. PHA-L4 Lectin Precipitation Assays

C6 and C6/BoDV-1 cells were grown to confluence and harvested from the culture dishes using a cell scraper. Cell lysates were prepared from equal numbers of cells (2.5 × 10^6^ cells per sample) in RIPA buffer containing protease inhibitor (cOmplete EDTA-free protease inhibitor cocktail tablets; Roche). The entire lysate corresponding to 2.5 × 10^6^ cells was incubated with PHA-L4 lectin-agarose (phytohemagglutinin L4-agarose; J-Oil Mills, Inc., Tokyo, Japan) at 4 °C overnight. The lectin-bound glycoprotein fractions were precipitated, collected, and subsequently analyzed by Western blotting with SAF32 or SAF83.

### 2.7. Cellular RNA Extraction and Reverse Transcription Polymerase Chain Reaction (RT-PCR)

Total RNA of cells was isolated using TRIzol reagent (Gibco BRL, Gaithersburg, MD, USA) by following the manufacturer’s instructions. cDNA was synthesized using a PrimeScript II 1st strand cDNA Synthesis kit (Takara Bio Inc., Otsu, Japan) under the following conditions: 65 °C for 5 min, 4 °C for 5 min, 42 °C for 60 min. Polymerase chain reaction (PCR) was performed with Ex Taq polymerase (Takara Bio Inc.). The PCR cycle parameters comprised an initial denaturation step of 95 °C for 5 min followed by 35 cycles of 95 °C for 30 s, 60 °C for 30 s, and 72 °C for 1 min, and then a final extension step of 72 °C for 10 min. PCR was carried out using the following oligonucleotide primers: for the MGAT1 gene, MGAT1-F: 5′-TTCTCTGTGGAGGCAGAGGT-3′ and MGAT1-R: 5′-ACGAACTGCTGGTTCAGCTT-3′; for the MGAT2 gene, MGAT2-F: 5′-CGCAGGGTTTCTAACGACTC-3′ and MGAT2-R: 5′-TGATATGCATCTCGGGTCAA-3′; for the MGAT3 gene, MGAT3-F: 5′-GGAGCCCAAGAGCACTGTAG-3′ and MGAT3-R: 5′-CCAGGCGTGTCTCCTTAGAG-3′; for the MGAT4 gene, MGAT4-F: 5′-ATAGCCAGTGCTCACCCAAC-3′ and MGAT4-R: 5′-CAAATGTCTTGAAGCCAGCA-3′; for the MGAT5 gene, MGAT5-F: 5′-CTAGGACCATCCTGGGTTCA-3′ and MGAT5-R: 5′-GGCAGAAGTCCTGTTTCTCG-3′; for the ETS1 gene, ETS1-F: 5′-ACAACGGCAGGAGCTAAGAA-3′ and ETS1-R: 5′-GCGTTTTCAGCATGAACTGA-3′; and for the glyceraldehyde 3-phosphate dehydrogenase (GAPDH) gene, G1: 5′-ACCACAGTCCATGCCATCAC-3′ and G2: 5′-TCCACCACCCTGTTGCTGTA-3′. PCR amplicons were analyzed by agarose gel electrophoresis (1.5% agarose gel). The amplified products were isolated and then verified by DNA sequencing (ABI PRISM3100 Genetic Analyzer; Applied Biosystems, Foster City, CA, USA). The intensity of the amplified bands from the PCR products was semi-quantitatively measured using ImageJ software 1.49v after agarose gel electrophoresis.

### 2.8. Viral RNA Extraction and RT-PCR

Viral RNA in the above fraction was extracted using a QIAamp Viral RNA mini kit (Qiagen, Hilden, Germany) according to the manufacturer’s instructions. cDNA was prepared from the extracted RNA using a PrimeScript II 1st strand cDNA Synthesis kit (Takara Bio Inc.) as follows. The eluted RNA was initially incubated at 65 °C for 5 min, and then the RNA was reverse-transcribed using random primers at 42 °C for 60 min, followed by denaturation of the enzyme at 95 °C for 5 min. The cDNA was amplified in a reaction mixture containing primers, Ex Taq (Takara Bio Inc.), and Ex Taq buffer under the following conditions: 25–35 cycles of 94 °C for 1 min, 62 °C for 1 min, and 72 °C for 1 min. PCR was carried out using the following previously reported oligonucleotide primers [46]: for the BoDV-1 N gene, BoDV-1 hu N-F: 5′-ATATGAACCCCCAGCGAGCCTCCCT-3′ and BoDV-1 hu N-R: 5′-TGCTCGGCTCCTGCTTTGATC-3′; for the BoDV-1 P gene, BoDV-1 hu p24-F: 5′-TCAGACCCAGACCAGCGAA-3′ and BoDV-1 hu p24-R: 5′-AGCTGGGGATAAATGCGCG-3′. PCR amplicons were analyzed by agarose gel electrophoresis using ImageJ.

### 2.9. Construction of GnTV Expression Plasmids and Establishment of Transfectants

The coding sequence of MGAT5 was amplified by PCR using gene-specific primers incorporating restriction enzyme recognition sites. The forward primer GnTV XhoI-F (5′-CTCGAGGCCACCATGGCTTTCTTTTCTCCCTGG-3′) and the reverse primer GnTV STOP HpaI-R (5′-GTTAACCTATAGGCAGTCTTTGCAGAG-3′) were used to generate the native GnTV construct. For the HA-tagged construct, the forward primer HA-GnTV XhoI-F (5′-CTCGAGGCCACCATGTACCCATACGACGTCCCAGACTACGCTGCTTTCTTTTCT-3′), which includes an N-terminal HA tag sequence, was used. The amplified PCR products were digested with XhoI and HpaI and ligated into the mammalian expression vector pCXN2, which had been linearized with XhoI and SmaI. The resulting plasmids encoded either untagged GnTV or N-terminally HA-tagged GnTV under the control of the CAG promoter (cytomegalovirus immediate-early enhancer/chicken β-actin promoter). All constructs were verified by DNA sequencing. After transfection of the plasmids, transfected C6 cells were selected in 10% FCS-DMEM containing G418 at 800 µg/mL. It should be noted that both GnTV and HA-GnTV constructs were used to examine the effect of GnTV overexpression and confirm reproducibility using independent constructs.

### 2.10. Flow Cytometry for PrP Detection

C6 or C6/BoDV-1 cells were harvested at three confluency levels (100%, 50%, and 25% confluent). The cells were stained on ice with either anti-PrP antibody SAF83 or SAF32, followed by fluorescein isothiocyanate (FITC)-conjugated anti-mouse IgG (H+L) antibody (Jackson ImmunoResearch). Fluorescence levels were analyzed using BD CellQuest Pro software (BD Biosciences, San Jose, CA, USA) and FACSCalibur (BD Biosciences). Intact cells were gated on the basis of forward scatter (FSC) and side scatter (SSC) to exclude cell debris and aggregates. An identical gating strategy was applied to both C6 and C6/BoDV-1 cells. Cells incubated with secondary antibody only were used as negative controls. For quantitative analysis, geometric mean (Geo Mean) and median fluorescence intensities were recorded for each sample. Background-corrected values were calculated by subtracting the corresponding secondary-antibody-only negative control value from the anti-PrP antibody-stained value for each condition. The ratio of corrected C6/BoDV-1 values to corrected C6 values was calculated to assess relative PrP expression levels between infected and uninfected cells.

### 2.11. Promoter Activity Assay

To assess ETS-dependent promoter activity, cells were transfected with the Trans-luc ETS reporter vector (Panomics Inc., Fremont, CA, USA) encoding a luciferase gene under the control of ETS-binding promoter elements (5′-GGAGGAGGGCTGCTTGAGGAAGTATAAGAATGGAGGAGGGCTGCTTGAGGAATATAAGAAT-3′) using the Nucleofector II device and nucleofection reagent (Amaxa Biosystems, Gaithersburg, MD, USA). After 24 h, luciferase activity was measured using a luciferase assay kit and a luminometer (Lumat LB9507; Berthold Technologies GmbH, Bad Wildbad, Germany).

### 2.12. SOD Activity Assays

Total SOD activity in cell lysates was measured using the SOD Assay Kit-WST (Dojindo, Kumamoto, Japan) in accordance with the manufacturer’s instructions. Absorbance at 450 nm was measured using a microplate reader, and activity (Units) was normalized to total protein. The protein concentration was measured using a Bio-Rad DC Protein Assay Kit (Bio-Rad).

### 2.13. Statistical Analyses

Statistical analyses were performed using GraphPad Prism (version 7.02; GraphPad Prism Software Inc., La Jolla, CA, USA). Differences were evaluated by unpaired *t*-test for a comparison between two groups, or by one-way analysis of variance (ANOVA) followed by Tukey’s multiple comparisons test for the comparison among three or more groups. A *p*-value < 0.05 was considered statistically significant. All data are shown as the average ± standard error of the mean (SEM).

## 3. Results

**Figure 1:**
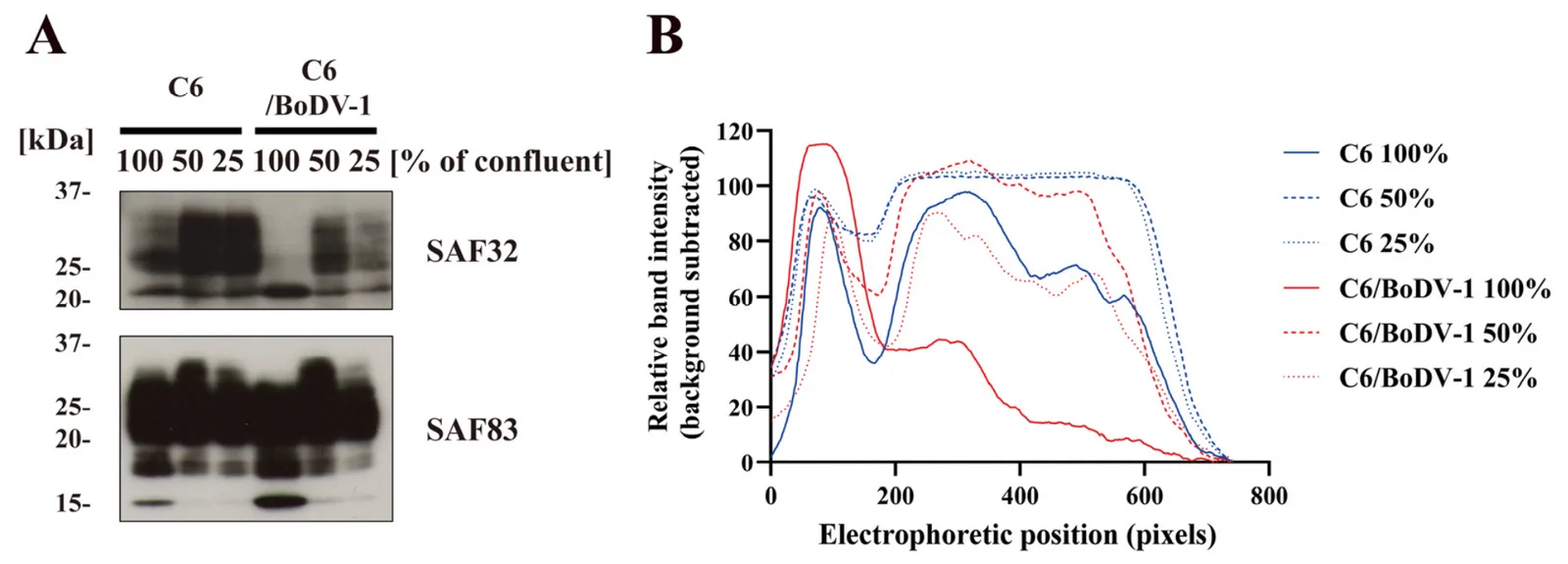
Cell density-dependent alteration of PrP glycoform migration is enhanced by Borna disease virus 1 (BoDV-1) infection. (**A**) C6 astroglial cells and BoDV-1-infected C6 cells (C6/BoDV-1) were cultured to 100%, 50%, or 25% confluency. The proteins (20 µg/lane) from the resultant cell lysates were subjected to Western blotting with anti-PrP (SAF32 and SAF83), as described in Materials and Methods. SAF32 recognizes the N-terminal region of PrP, while SAF83 recognizes the C-terminal half of PrP. The proportion of PrP with a lower molecular weight appeared to increase with increasing cell density. Moreover, this phenomenon was particularly marked for C6/BoDV-1 cells compared to C6 cells at 100% confluency. The locations of molecular weight markers (kDa) are shown on the left-hand side of each blot. (**B**) Densitometric lane profiles of the SAF32 immunoblot shown in Figure 1A. Intensity profiles along the migration axis were obtained for each lane using ImageJ plot profile analysis. Background correction was performed by subtracting the minimum pixel intensity within each lane. The *x*-axis represents electrophoretic position within the analyzed molecular weight range (expressed in pixels). Based on co-migrating molecular weight markers, pixel 0 and pixel 743 correspond to approximately 20 kDa and 37 kDa, respectively. C6 cells (blue) and C6/BoDV-1 cells (red) at 100% (solid line), 50% (dashed line), and 25% (dotted line) confluency are shown. In C6/BoDV-1 cells at 100% confluency, the PrP signal was distributed toward a lower electrophoretic position, corresponding to a lower molecular weight, consistent with an accumulation of lower-molecular-weight glycoforms. Data from the complete confluency series shown in Figure 1A are from one representative experiment for SAF32 and one representative experiment for SAF83.

Initially, BoDV-1 infection in C6/BoDV-1 cells was confirmed by Western blotting using anti-BoDV-1 p40 antibody (Figure S1). Next, we examined whether BoDV-1 infection affects the electrophoretic profile of PrP in rat astroglial C6 cells. Western blotting using SAF32 and SAF83 showed that PrP in uninfected C6 cells migrated as multiple bands corresponding to different glycoforms. This broad immunoreactive pattern is consistent with the known heterogeneity of PrP *N*-glycosylation. Specifically, PrP contains two *N*-linked glycosylation sites and is expressed as di-, mono-, and unglycosylated glycoforms. The relative abundance of the lower-molecular-weight bands appeared to increase as the cell density increased from 25% to 100% confluency (Figure 1A). This tendency was more evident in C6/BoDV-1 cells than in C6 cells, particularly at 100% confluency. Indeed, at 100% confluency of C6/BoDV-1 cells, PrP displayed a higher proportion of lower-molecular-weight species compared with C6 cells. Densitometric lane profiles of the SAF32 immunoblot, corresponding to a molecular mass range of 20 kDa to 37 kDa, were analyzed. The densitometric lane profile indicates that the PrP signal was concentrated at a lower electrophoretic position in C6/BoDV-1 cells, particularly at 100% confluency, whereas C6 cells showed a broader distribution extending toward higher molecular weight positions (Figure 1B). A similar directional shift was qualitatively observed in the SAF83 immunoblot. Overall, these observations are consistent with altered PrP glycoform migration in C6/BoDV-1 cells, which reflects altered glycosylation rather than a change in total PrP expression.

**Figure 2:**
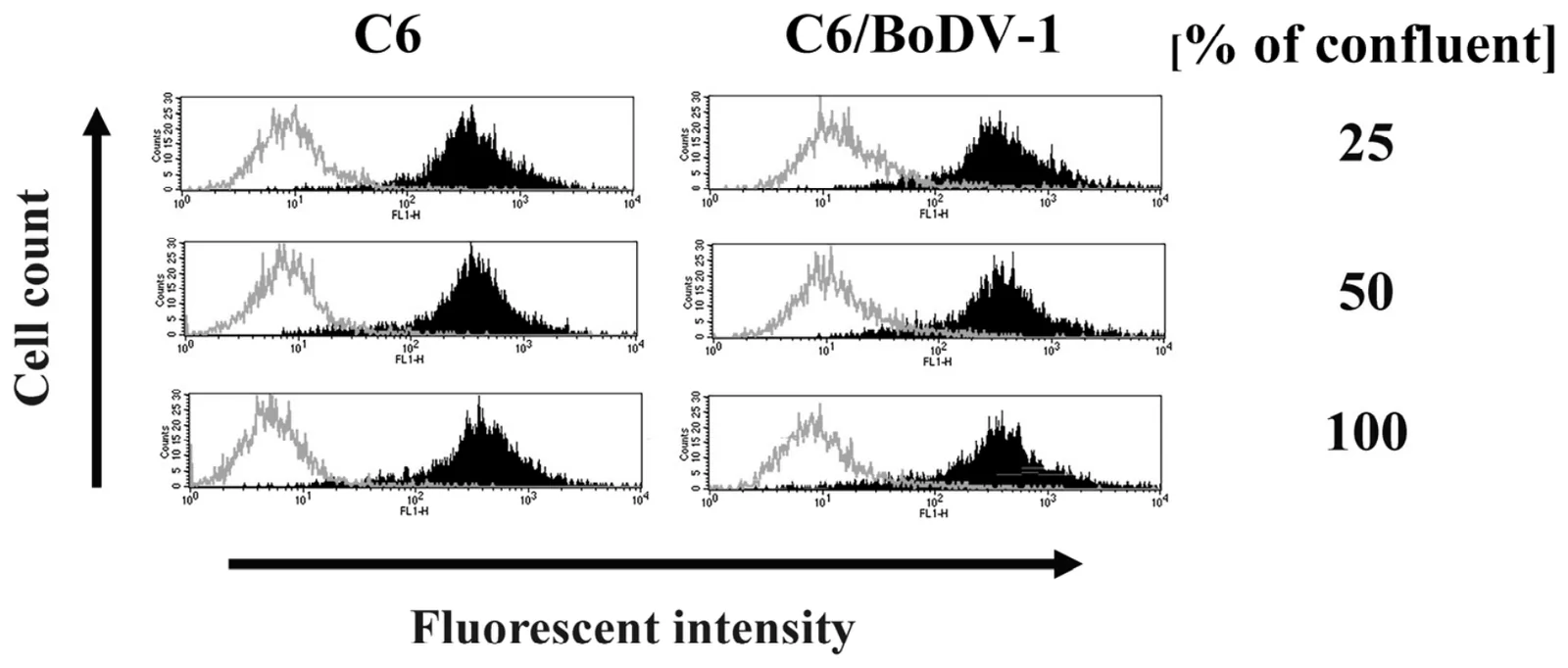
The expression level of PrP remains unchanged in C6 cells cultured to different confluency or following BoDV-1 infection. C6 cells and those infected with BoDV-1 (C6/BoDV-1) were subjected to flow cytometry using anti-PrP antibody SAF83. In each panel, filled histograms represent PrP-stained cells and open histograms represent the negative control. Fluorescence intensity profiles revealed no major differences in PrP expression between C6 and C6/BoDV-1 cells at any cell density. Data from one representative flow-cytometric experiment using SAF83 are shown. An additional flow-cytometric analysis was also performed using SAF32 and showed a similar trend.

Next, to determine whether BoDV-1 infection or cell density influenced total PrP expression at the cell surface, we analyzed C6 and C6/BoDV-1 cells by flow cytometry using anti-PrP antibody SAF83. Fluorescence intensity profiles of PrP staining were comparable between C6 and C6/BoDV-1 cells at 100%, 50% and 25% confluency (Figure 2). Background-corrected Geo Mean and Median values of fluorescent intensity in the flow-cytometric analysis showed C6/BoDV-1-to-C6 ratios of 0.95–1.02 and 0.97–0.99, respectively, across all confluency conditions, showing that total surface PrP expression is not substantially altered by BoDV-1 infection (Table 1). Together, these results indicate that BoDV-1 infection and reduced cell density do not substantially alter total PrP expression at the cell surface. Instead, the glycosylation pattern of PrP is modified, resulting in the accumulation of lower-molecular-weight glycoforms.

**Figure 3:**
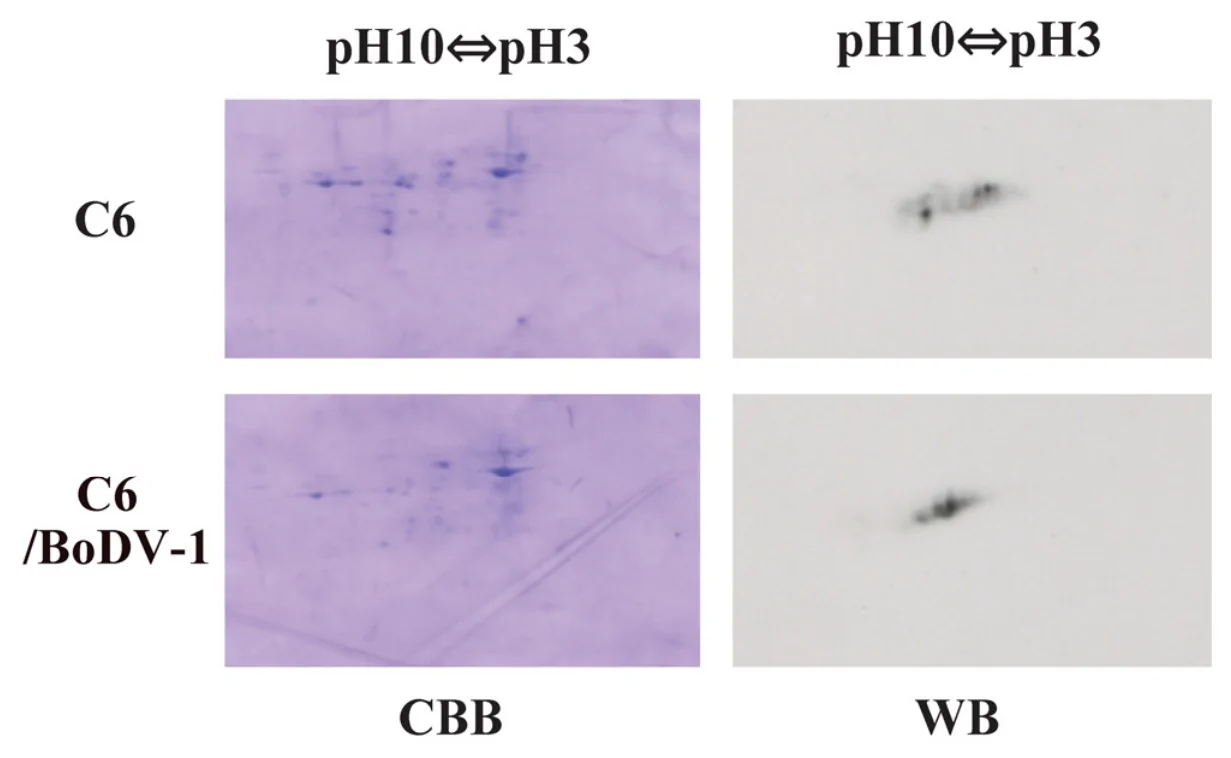
PrP in BoDV-1 infected cells displays a lower molecular weight and more basic isoelectric point than PrP in C6 cells. Confluent monolayers of C6 and C6/BoDV-1 cells were harvested using a scraper. The extracted samples prepared from equal numbers of C6 and C6/BoDV-1 cells (2.5 × 10^6^ cells/sample) were subjected to two-dimensional electrophoresis (i.e., isoelectric focusing (pH 3–10) followed by SDS-PAGE). Coomassie Brilliant Blue staining (CBB) and Western blotting with SAF32 (WB) are shown. In C6/BoDV-1 cells, PrP spots shifted to a lower molecular weight and more basic isoelectric point compared with C6 cells. It should be noted that the most acidic, highly glycosylated PrP species detected in C6 cells were markedly diminished or absent in C6/BoDV-1 cells. In the panels, the horizontal axis (isoelectric focusing dimension) is oriented from pH 10 on the left-hand side to pH 3 on the right-hand side. Although molecular weight markers were not run in the SDS-PAGE (vertical) dimension, the molecular weights of the PrP species can be estimated from the one-dimensional electrophoresis performed under the same conditions and detected with the same SAF32 antibody (Figure 1A), in which the PrP signal corresponds to approximately 20–37 kDa. Data from one representative 2D-PAGE experiment using SAF32 are shown. A similar 2D-PAGE analysis was also performed using SAF83 and showed a similar trend.

Next, 2D-PAGE followed by immunoblotting using anti-PrP antibody SAF32 further clarified the biochemical nature of the PrP species. PrP from C6 cells resolved into several spots differing in both molecular weight and isoelectric point, consistent with heterogeneous *N*-glycosylation (Figure 3). In C6/BoDV-1 cells, PrP spots shifted toward lower molecular weight and more basic isoelectric points. Notably, the most acidic, slowly migrating spot observed in C6 cells was either markedly reduced or absent in C6/BoDV-1 cells. These findings indicate that BoDV-1 infection interferes with the maturation of complex *N*-glycans on PrP and favors under-glycosylated forms.

**Figure 4:**
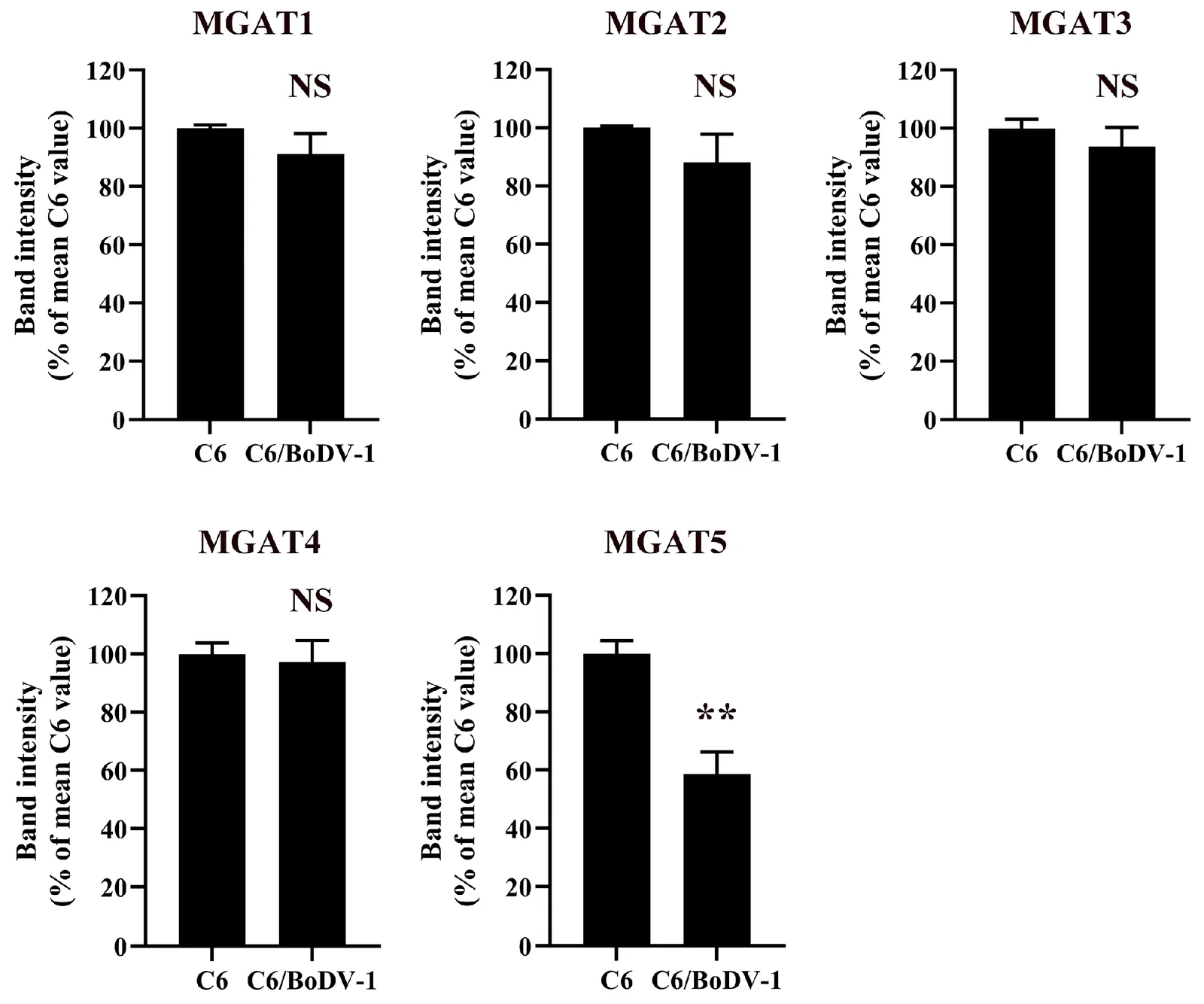
Reduced RT-PCR band signal of the MGAT5 gene encoding *N*-acetylglucosaminyltransferase V (GnTV) in BoDV-1-infected C6 cells. Total RNA was extracted from C6 cells and BoDV-1-infected C6 cells (C6/BoDV-1) collected at 100% confluency on three independent days. These samples are referred to as three independently prepared RNA samples (*n* = 3). The RNA samples were reverse-transcribed, and polymerase chain reaction (PCR) was used to amplify *N*-acetylglucosaminyltransferase I (GnTI) encoding gene MGAT1, *N*-acetylglucosaminyltransferase II (GnTII) encoding gene MGAT2, *N*-acetylglucosaminyltransferase III (GnTIII) encoding gene MGAT3, *N*-acetylglucosaminyltransferase IV (GnTIV) encoding gene MGAT4, as well as MGAT5. PCR band intensities were quantified using ImageJ. For each gene, the mean band intensity of the three C6 samples was defined as 100%, and individual values from both C6 and C6/BoDV-1 samples were expressed as percentages of this mean reference value. Thus, the 100% value represents the mean of the C6 reference group, whereas the error bar indicates variability among the three independent C6 and C6/BoDV-1 samples. RT-PCR band signal for MGAT5 was lower in C6/BoDV-1 cells than in C6 cells, whereas the signals of MGAT1, MGAT2, MGAT3, and MGAT4 were not markedly altered between C6 and C6/BoDV-1 cells. Data (*n* = 3) are shown as mean ± SEM. ** *p* < 0.01: significant difference compared to the uninfected counterpart. NS: no significant difference compared to the uninfected counterpart.

Next, to investigate whether the BoDV-1-induced alteration of PrP glycosylation was associated with changes in *N*-acetylglucosaminyltransferase expression, we analyzed the mRNA levels of MGAT1-5 as well as the BoDV-1 N and P genes by RT-PCR. In C6/BoDV-1 cells, a reduction in the MGAT5 RT-PCR signal was observed compared with C6 cells, whereas MGAT1-4 showed only slight or negligible changes (Figure 4). BoDV-1 N and P transcripts were present only in C6/BoDV-1 cells, confirming persistent infection (Figure S2).

**Figure 5:**
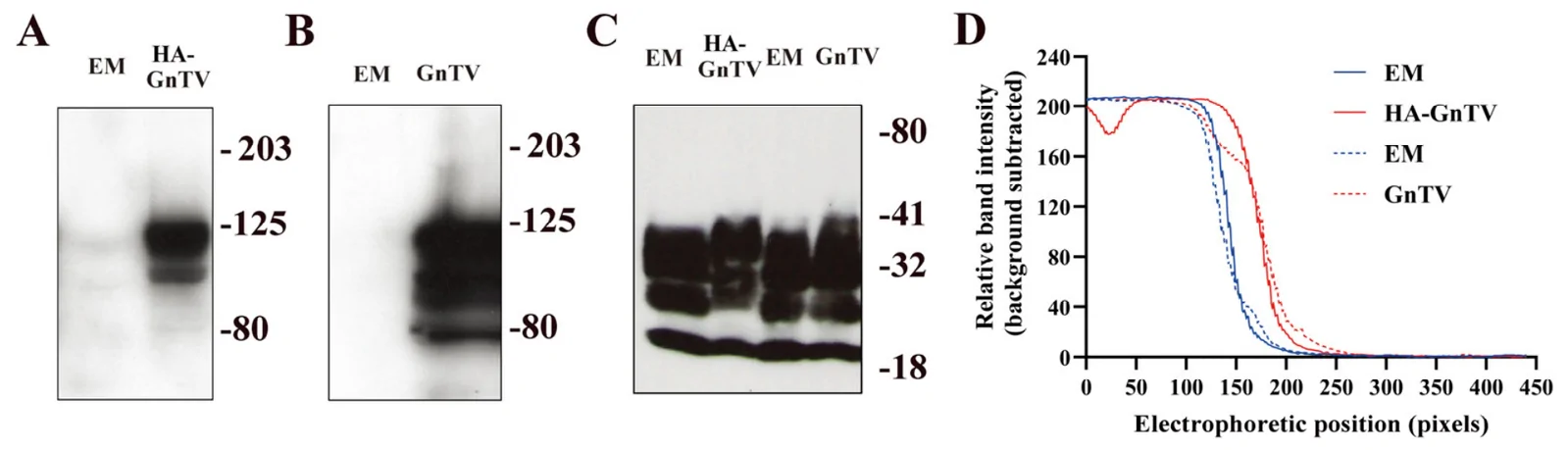
Overexpression of GnTV is associated with a shift of PrP to higher-molecular-weight glycoforms. C6 cells were transfected with either an empty vector (EM), a GnTV/MGAT5 expression vector (GnTV), or an HA-tagged GnTV/MGAT5 expression vector (HA-GnTV). (**A**) Western blotting with anti-GnTV antibody (24D11) showed HA-GnTV overexpression in HA-GnTV-transfected cells compared with EM controls. (**B**) Western blotting with anti-GnTV antibody (24D11) showed GnTV overexpression in GnTV-transfected cells compared with EM controls. (**C**) Western blotting with SAF83 showed an upward shift of PrP bands in HA-GnTV- and GnTV-overexpressing cells. The locations of molecular weight markers (kDa) are shown on the right-hand side of each blot. (**D**) Densitometric lane profiles of the SAF83 immunoblot shown in Figure 5C. Intensity profiles along the migration axis were obtained for each lane using ImageJ plot profile analysis. Background correction was performed by subtracting the minimum pixel intensity within each lane. The x-axis represents electrophoretic position (pixels). Based on co-migrating molecular weight markers, pixel 0 and pixel 441 correspond to approximately 32 kDa and 80 kDa, respectively. EM (blue line and blue dots) and HA-GnTV (red line) as well as GnTV (red dots) are shown. In HA-GnTV- and GnTV-overexpressing C6 cells, the PrP signal was distributed toward a higher electrophoretic position, corresponding to higher molecular weights, compared to the control counterpart (EM). These observations are consistent with an increased proportion of higher-molecular-weight PrP glycoform species. Data from one representative immunoblot experiment using SAF83 are shown. An additional immunoblot analysis was also performed using SAF32, which displayed a similar trend.

To further directly examine the impact of GnTV on PrP glycosylation, we overexpressed GnTV or HA-tagged GnTV in C6 cells. Western blotting with the 24D11 anti-GnTV antibody showed robust expression of GnTV in GnTV- and HA-GnTV–transfected cells, whereas only a faint endogenous band was detected in empty vector (EM) controls (Figure 5A,B). When PrP was analyzed with SAF83, C6 cells overexpressing GnTV or HA-GnTV showed an upward shift of PrP bands compared with EM controls, with an increased proportion of slower-migrating, higher-molecular-weight species (Figure 5C). Note that both constructs produced a similar upward shift in the PrP bands. Thus, the observed shift in migration was reproducible using independent GnTV constructs. Furthermore, densitometric lane profiles of the SAF83 immunoblot, corresponding to the molecular mass range of approximately 32 kDa to 80 kDa, showed that the relative band intensity signal of highly glycosylated PrP shifted to a higher electrophoretic position in C6 cells overexpressing GnTV or HA-tagged GnTV, compared to empty vector (EM) counterparts (Figure 5D). These observations indicate that GnTV overexpression enhances the addition of complex *N*-glycans, thereby increasing the molecular weight of PrP.

**Figure 6:**
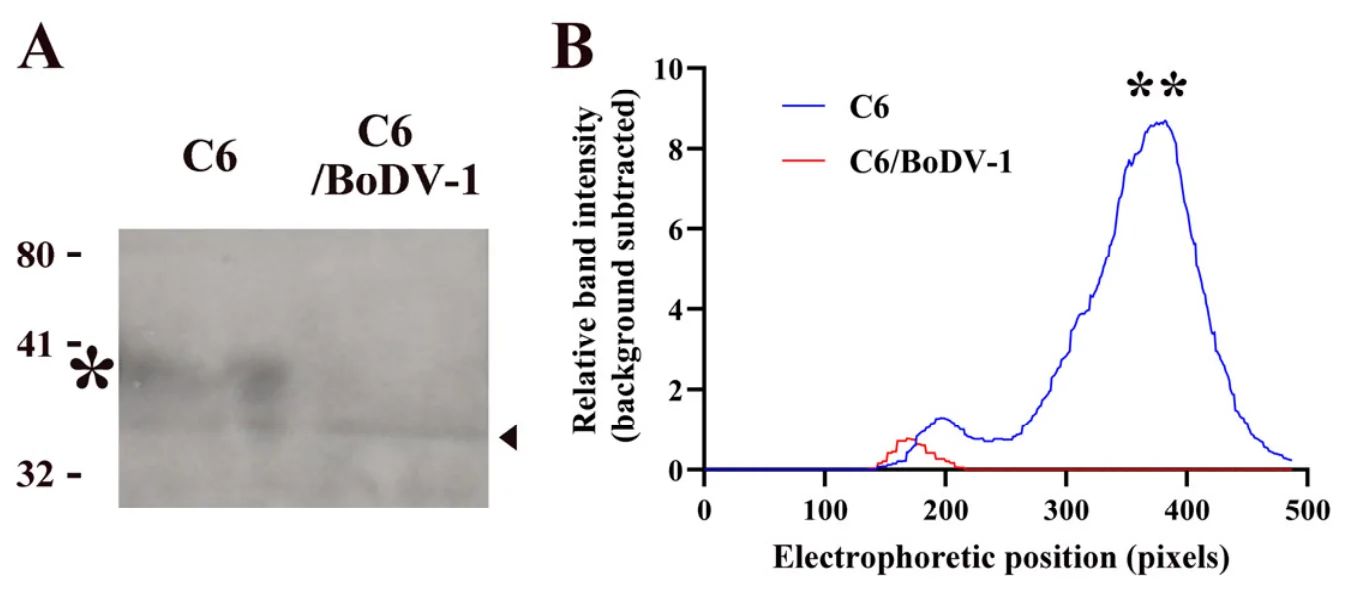
Reduced PHA-L4-reactive PrP signal after BoDV-1 infection. (**A**) Confluent monolayers of C6 and C6/BoDV-1 cells were harvested using a scraper. PHA-L4 lectin-agarose, which binds to β1-6-branched tri- and tetra-antennary *N*-glycans, was added and the precipitate collected. The precipitants were subjected to Western blotting with anti-PrP SAF32. C6 cells exhibited PHA-L4-reactive PrP signals at around 35~40 kDa (*), whereas this signal was not clearly detected in C6/BoDV-1 cells. The arrowhead (◀) highlights commonly observed bands in both C6 and C6/BoDV-1 that are considered non-specific. The locations of molecular weight markers (kDa) are shown on the left-hand side of the blot. (**B**) Densitometric lane profiles of the band intensity shown in Figure 6A. Intensity profiles along the migration axis were obtained for each lane using ImageJ plot profile analysis. Background correction was performed by subtracting the minimum pixel intensity within each lane. The x-axis represents electrophoretic position (pixels). Based on co-migrating molecular weight markers, pixel 0 and pixel 487 correspond to approximately 32 kDa and 41 kDa, respectively. C6 cells (blue) and C6/BoDV-1 cells (red) are shown. In C6/BoDV-1 cells, the signal intensity at around 35~40 kDa (**) appeared reduced compared with C6 cells. Data from one representative PHA-L4 precipitation experiment followed by SAF32 immunoblotting are shown. An additional PHA-L4 precipitation analysis followed by SAF83 immunoblotting was also performed, which showed a similar trend.

To further determine whether BoDV-1 infection affects the *N*-glycosylation status of PrP, PrP species carrying β1-6-branched tri- and tetra-antennary *N*-glycans were precipitated by PHA-L4 lectin-agarose and further analyzed by Western blotting using the anti-PrP antibody SAF32. In uninfected C6 cells, PHA-L4-reactive PrP signals were detected at approximately 35–40 kDa (Figure 6A). No such signals were observed in C6/BoDV-1 cells, indicating a marked reduction in PHA-L4-reactive *N*-glycosylated PrP following BoDV-1 infection. Furthermore, densitometric analysis of the lane profiles, corresponding to a molecular mass range of approximately 32 kDa to 41 kDa, showed that the relative band intensity signal of C6/BoDV-1 cells at around 35~40 kDa was decreased compared to C6 cells (Figure 6B). These results indicate that BoDV-1 infection decreases the abundance of PrP species bearing β1-6-branched complex *N*-glycans.

**Figure 7:**
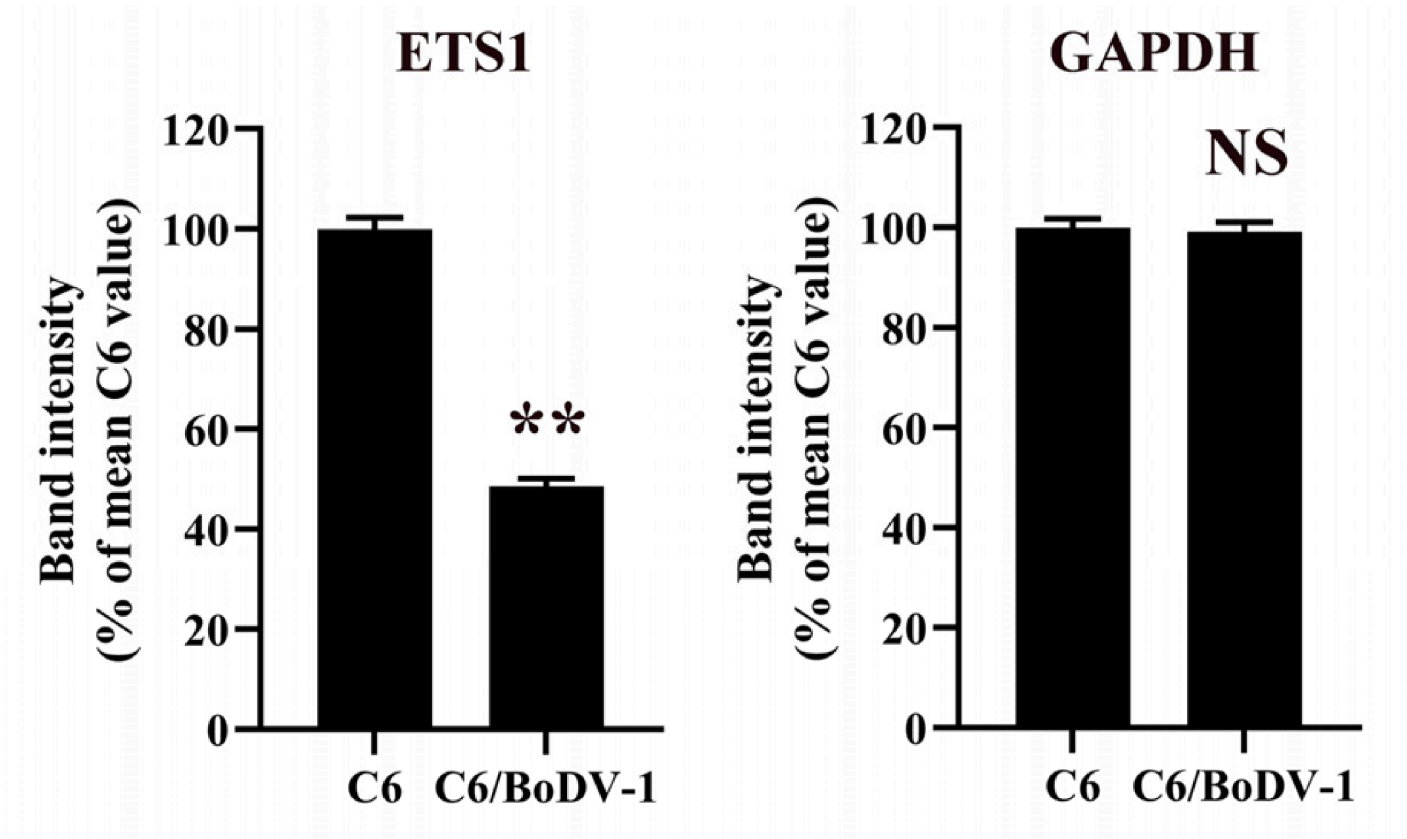
Reduced ETS1 RT-PCR signal in BoDV-1-infected C6 cells. Total RNA was extracted from C6 cells and BoDV-1-infected C6 cells (C6/BoDV-1) collected at 100% confluency on three independent days. These samples are referred to as three independently prepared RNA samples (*n* = 3). The RNA samples were reverse-transcribed, and PCR was used to amplify the ETS1 gene and glyceraldehyde-3-phosphate dehydrogenase (GAPDH) fragments. PCR band intensities were quantified using ImageJ. For each gene, the mean band intensity of the three C6 samples was defined as 100%, and individual values from both C6 and C6/BoDV-1 samples were expressed as percentages of this mean reference value. Thus, the 100% value represents the mean of the C6 reference group, whereas the error bar indicates the variability among the three independent C6 and C6/BoDV-1 samples. A reduction in the ETS1 RT-PCR signal was observed in C6/BoDV-1 cells compared to C6 cells, while GAPDH RT-PCR signals remained constant. Data (*n* = 3) are shown as mean ± SEM. ** *p* < 0.01: significant difference compared to the uninfected counterpart. NS: no significant difference compared to the uninfected counterpart.

**Figure 8:**
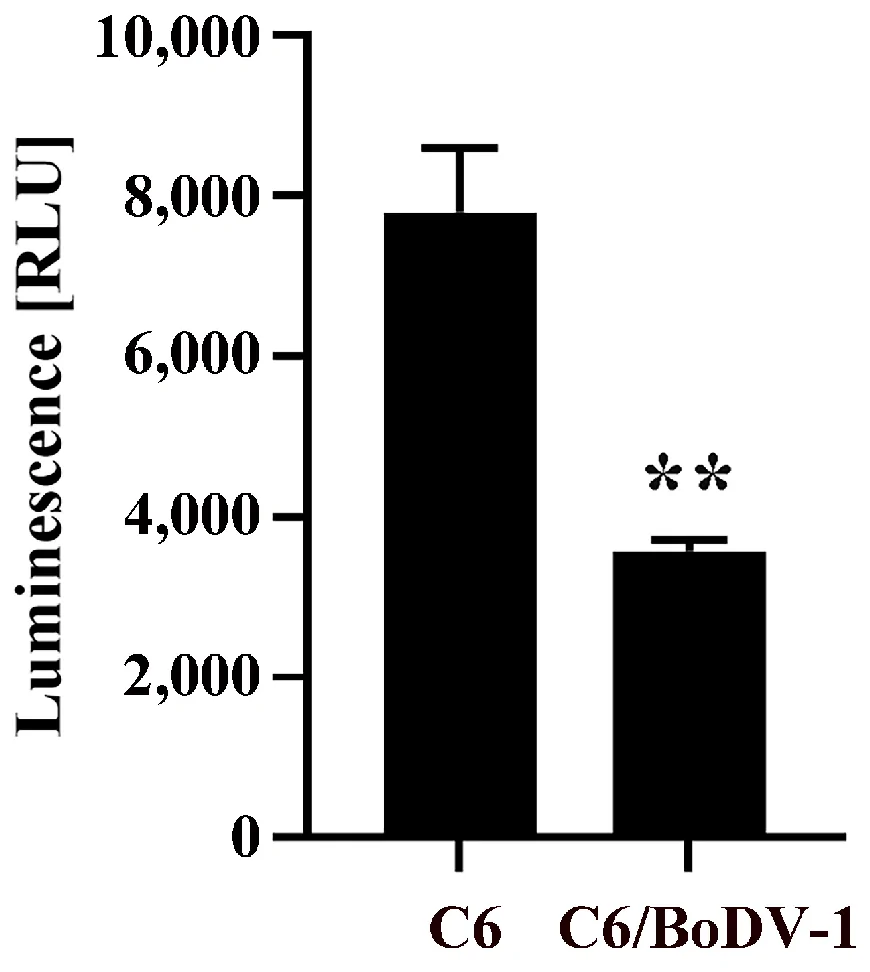
BoDV-1 infection suppresses ETS-dependent promoter activity. C6 and C6/BoDV-1 cells were transiently transfected with a Trans-luc ETS reporter vector containing ETS-binding sites upstream of a luciferase gene. After incubation, luciferase activity was measured using a luminometer and expressed in relative light units (RLU). Reporter activity was significantly reduced in C6/BoDV-1 cells compared with C6 cells. Data are shown as mean ± SEM from nine independently prepared cell lysates (*n* = 9). ** *p* < 0.01: significant difference compared to the uninfected counterpart.

The MGAT5 promoter contains ETS-binding elements [29]. Thus, we next examined whether BoDV-1 infection affects the expression of the transcription factor Ets-1. RT-PCR analysis showed a lower ETS1 band signal in C6/BoDV-1 cells compared to C6 cells, while the GAPDH band signal remained unchanged (Figure 7). To assess the functional consequences of this decrease in Ets-1, we performed a luciferase reporter assay using a Trans-luc ETS vector containing ETS-binding sites upstream of the luciferase gene. Reporter activity was significantly lower in C6/BoDV-1 cells than in C6 cells (Figure 8). These results indicate that BoDV-1 infection is associated with a reduced ETS1 RT-PCR signal and ETS-dependent promoter activity, which may contribute to reduced MGAT5 expression and impaired complex *N*-glycan branching on PrP.

**Figure 9:**
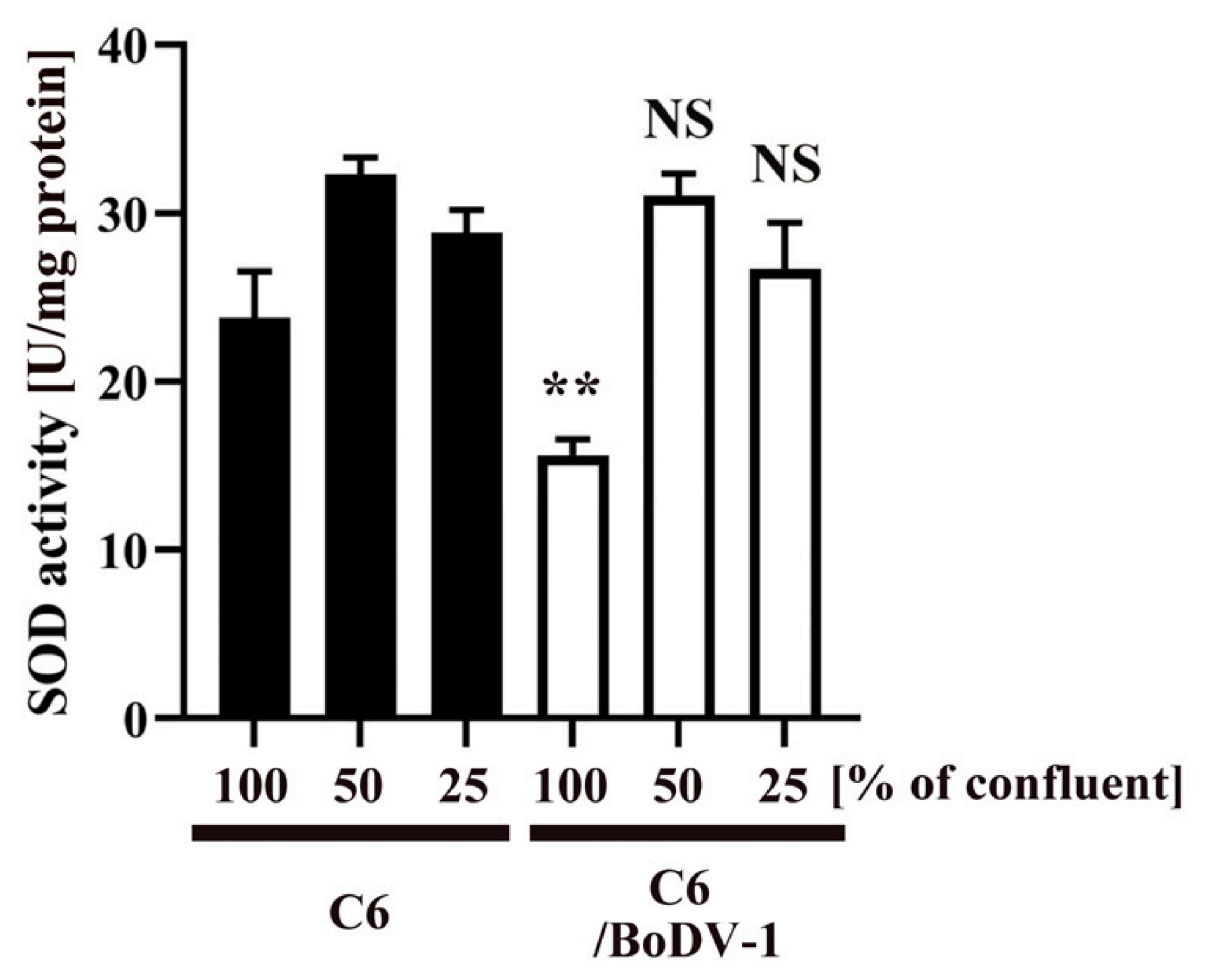
BoDV-1 infection decreases cellular superoxide dismutase (SOD) activity. C6 and C6/BoDV-1 cells were harvested at 100%, 50%, or 25% confluency, and total SOD activity in cell lysates was measured using a SOD assay kit-WST (Dojindo). Results are expressed as SOD activity (Units/mg protein). BoDV-1-infected cells showed significantly reduced SOD activity at 100% confluency relative to C6 cells. Data are shown as mean ± SEM from 6~10 independently prepared cell lysates for each condition (*n* = 6~10). ** *p* < 0.01: significant difference compared to the uninfected counterpart. NS: no significant difference compared to the uninfected counterpart.

Finally, we examined whether the BoDV-1-induced alteration in PrP glycosylation and GnTV expression has functional consequences for cellular redox homeostasis. Total SOD activity (Units/mg protein) was measured in C6 and C6/BoDV-1 cells at different levels of confluency. In C6/BoDV-1 cells, SOD activity was significantly reduced at 100% confluency compared to the uninfected C6 cells (Figure 9).

## 4. Discussion

Protein glycosylation is a fundamental post-translational modification that influences protein folding, trafficking, stability, and signaling. Our observations in rat astroglial C6 cells infected with BoDV-1 suggested that BoDV-1 infection alters the PrP glycoform profile, with a shift toward under-glycosylated PrP species despite unchanged overall levels of PrP. The BoDV-1-induced low-glycosylation of PrP may affect PrP function and/or cellular vulnerability. Moreover, astrogliosis is characteristic of BoDV-1 persistence in the central nervous system [35], raising the possibility that virus-glycosylation interactions in astrocytes could affect astroglial stress responses. This idea is consistent with evidence that persistent BoDV-1 infection can induce sustained host-cell alterations [35,36,37,38,41,42]. The CRISPR/Cas13b system has been used to reduce viral mRNAs and genomic RNA in persistently infected cells, suggesting that the inhibition of BoDV-1 replication and genome can be targeted by antiviral treatments [48]. The present study extends these concepts by showing the relationships of a virus-sensitive transcriptional axis (ETS1-MGAT5) that regulates *N*-glycan maturation of PrP in astroglial cells and by potentially linking this glycan remodeling to impaired antioxidative capacity during persistent BoDV-1 infection.

A key observation is that persistent BoDV-1 infection profoundly alters PrP electrophoretic mobility and 2D-PAGE distribution with essentially no change in total surface PrP levels. In uninfected C6 cells, increased confluency was associated with a shift toward faster-migrating (lower-molecular-weight) PrP species. However, BoDV-1 infection markedly enhanced this observed shift even at high confluency. Moreover, 2D-PAGE analysis revealed that BoDV-1 infection induces a redistribution toward lower molecular weight PrP species with more basic isoelectric points, with an almost complete loss of the most acidic, slowly migrating PrP species. These results indicate that BoDV-1 modifies the “quality” of PrP (i.e., its glycoform maturation) rather than its abundance.

PHA-L4 lectin precipitation followed by anti-PrP immunoblotting showed a reduction in PrP species bearing β1-6-branched complex *N*-glycans in BoDV-1-infected cells. Because PHA-L4 preferentially recognizes GnTV-dependent tri-/tetra-antennary structures, the reduction in PHA-L4 reactive PrP signal is consistent with impaired GnTV-dependent branching rather than a total absence of *N*-glycosylation. This interpretation is supported by the RT-PCR results showing that band signals of MGAT5 (but not MGAT1-4) were reduced in BoDV-1-infected cells, supporting the depletion of GnTV expression. Collectively, the data identify selective suppression of GnTV as the probable mechanism underlying the loss of β1-6 branching and the accumulation of under-glycosylated PrP isoforms.

This conclusion is strengthened by functional manipulation. GnTV overexpression (including HA-GnTV) shifted PrP to higher-molecular-weight glycoforms. Thus, GnTV overexpression is sufficient to promote the emergence of higher-molecular-weight PrP species with more fully branched complex *N*-glycans in this astroglial system.

The mechanistic link between BoDV-1 infection and MGAT5 suppression is supported by concordant decreases in ETS1 RT-PCR signal and ETS-dependent reporter activity. Prior work had established that the MGAT5 promoter contains Ets-binding elements and that Ets-1 positively regulates MGAT5 transcription [29]. Our data are consistent with this model and suggest that BoDV-1 represses MGAT5, at least in part, through attenuation of ETS1 expression, thereby reducing Ets-dependent promoter activation. BoDV-1 is known to induce broad, persistent host transcriptional alterations [37,41,42] and can interact with host chromatin-associated processes [38,41,42]. Thus, transcriptional reprogramming may represent a plausible and potential upstream mechanism to explain these observations. Whether ETS1 suppression is triggered by specific viral gene products, host stress-signaling, or epigenetic changes remains an important question for future work.

Taken together, our results suggest BoDV-1 infection leads to a qualitative impairment of PrP function associated with altered glycan maturation rather than reduced PrP expression. The subsequent loss of complex branching likely changes PrP conformation, trafficking, or membrane microdomain partitioning. These changes may lead to an alteration in the ability of PrP to engage with cytoprotective partners and signaling pathways. Notably, PrP localization to lipid rafts has been linked to its signaling context and trafficking behavior [49]. Membrane topology and trafficking features have been reported to influence PrP glycosylation efficiency and glycoform outcomes [50]. In this context, BoDV-1-driven suppression of GnTV may shift PrP to an underglycosylated form that is potentially less competent at supporting PrP-dependent antioxidative functions.

*N*-glycan branching is regulated by cellular metabolism, especially UDP-GlcNAc availability, and changes in nutrient conditions can modulate glycan complexity and glycoprotein surface retention [51,52]. Thus, the intrinsic cell density-dependent PrP band shift in uninfected C6 cells may reflect growth-state and metabolic regulation of branching capacity, while BoDV-1 amplifies this baseline plasticity via transcriptional suppression of ETS1 and MGAT5. Consistent with this view, BoDV-1 infection has been reported to perturb cellular metabolic profiles, including energy metabolites and amino acids [53], which could further bias glycan processing and redox balance.

From the perspective of viral neuropathogenesis, BoDV-1 is unusual in establishing persistent, largely noncytolytic infection in neural cells while producing broad and durable host regulatory changes [38]. The present work identifies the ETS1-MGAT5 axis as a specific host pathway that is suppressed during persistent BoDV-1 infection, which alters the glycosylation landscape of astroglia. Given the central role of astrocytes in maintaining redox homeostasis and supporting neuronal survival, potential impairment of astroglial antioxidative capacity may provide a plausible mechanism contributing to chronic neural dysfunction during persistent infection.

Supporting this hypothesis, BoDV-1-infected cells exhibited decreased cellular SOD activity. This finding is consistent with the concept that persistent infection drives a shift toward oxidative stress. PrP has been linked to cellular redox homeostasis and antioxidative defenses, including modulation of SOD activity and metal metabolism [54]. Indeed, oxidative stress is increasingly recognized as a central pathological contributor in prion diseases [11,55,56]. In addition, previous studies have shown that cellular redox status is closely linked to the metabolic state and is dynamically regulated by antioxidative function systems [57,58]. Given that total PrP levels were unchanged by flow cytometry, our results point to a qualitative impairment of PrP function associated with altered *N*-glycan maturation rather than reduced PrP expression. One potential mechanism is that loss of complex branching changes PrP conformation, trafficking, or membrane microdomain partitioning, thereby altering its ability to engage cytoprotective partners and/or signaling pathways. Notably, PrP localization to lipid rafts has been linked to its signaling context and trafficking behavior [49]. Membrane topology and secretory trafficking features can influence PrP glycosylation efficiency and glycoform outcomes [50]. Consequently, BoDV-1-driven suppression of GnTV may shift PrP to a glycoform repertoire that is less capable of supporting PrP-dependent antioxidative functions, contributing to the observed reduction in SOD activity.

In this study, several limitations should be emphasized. Firstly, although rat C6 cells provide a reproducible model of persistent BoDV-1 infection, the present findings were obtained using a single rat astroglial cell line. To assess the generalizability of our results, it is important to confirm these findings in primary astrocytes and neurons, as well as using *in vivo* infection models. In addition, their applicability to human astrocytes remains to be established. Although basic features of PrP are conserved in mammals, its glycoform processing may differ among species and cell types. Thus, the findings of this study need to be verified using primary human astrocytes or human induced pluripotent stem cell-derived astrocytes. Furthermore, *in vitro* confluence does not directly reflect astrocyte density *in vivo* but represents changes in cell–cell contact, growth state, membrane organization, and metabolism that may affect *N*-glycan processing. Thus, the observed PrP shift may model some conditions of proliferative *versus* mature or reactive astroglial states, although this interpretation requires validation in primary astrocytes and infected brain tissues. Secondly, although lectin precipitation may support loss of β1-6 branching, direct structural characterization of *N*-glycans of PrP by mass spectrometry-based glycoproteomics will be needed to define precisely which branches and terminal modifications are altered. In addition, we did not directly demonstrate that ETS1 regulates the MGAT5 promoter in this system, which would require chromatin immunoprecipitation, ETS1 knockdown/overexpression, or promoter mutagenesis to establish this causal link. Moreover, densitometric comparison of RT-PCR bands is semi-quantitative, limiting rigorous quantitative interpretation. Therefore, the RT-PCR data should be interpreted as supportive evidence for altered transcript detection, rather than as definitive quantitative measurement of mRNA abundance. Further quantitative analysis of ETS1 and MGAT5, such as quantitative RT-PCR, will be required. In addition, although change of GnTV expression level is sufficient to induce the PrP band shift, other glycosylation steps may also contribute to the antioxidative phenotype. Although reduced SOD activity accorded with altered PrP *N*-glycan maturation in BoDV-1-infected cells, the present data are correlative. The data are not sufficient to establish that PrP glycan remodeling causes a reduction in SOD activity. For instance, BoDV-1 may also affect SOD activity through PrP-independent pathways. Further experiments should be performed by restoring MGAT5/GnTV expression in C6/BoDV-1 cells to investigate whether both PHA-L4-reactive PrP glycoforms and SOD activity or resistance to oxidative stress are rescued. Loss-of-function experiments such as MGAT5 knockdown would determine whether reduced *N*-glycan branching is sufficient to reduce the SOD activity. Furthermore, the reintroduction of wild-type PrP or *N*-glycosylation-site mutants into PrP-gene-deficient cells, such as HpL3-4 cells [25,26,27], would help establish whether the effect is specifically dependent on PrP glycosylation. In addition, it should be noted that PrP glycan remodeling might affect functions beyond SOD regulation, including STI1-dependent signaling and copper binding/chelation. The potential impact on glycosylation-dependent PrP functions remains unclear from the present study. Although the major region of STI1- and copper-binding sites is located in the N-terminal domain, altered *N*-glycan maturation in the C-terminal domain might indirectly modify these functions by affecting PrP conformation, trafficking, or localization. In this regard, future studies using GnTV rescue or PrP *N*-glycosylation-site mutants are warranted.

## 5. Conclusions

**Figure 10:**
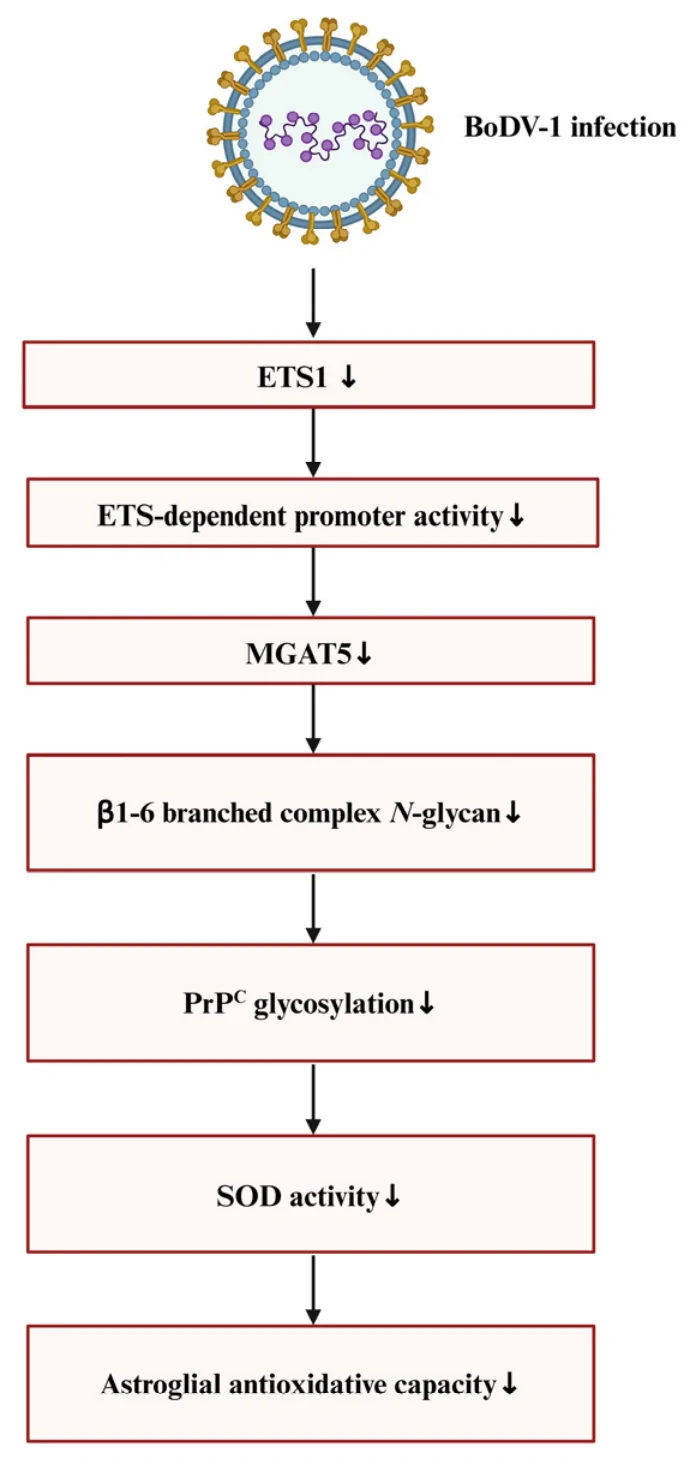
Schematic presentation of a proposed model of PrP^C^ glycosylation regulation by the GnTV-Ets1 axis in rat astroglial C6 cells. Persistent BoDV-1 infection may be associated with a reduced ETS1 RT-PCR signal, decreased ETS-dependent promoter activity, and reduced MGAT5/GnTV-related β1-6-branched complex *N*-glycan signals. These changes may contribute to a shift of PrP^C^ toward lower-molecular-weight glycoform species. These proposed changes appear to be associated with decreased SOD activity, suggesting potential implications for impaired astroglial antioxidative capacity. Created in BioRender. Sakudo, A. (2026) https://BioRender.com/lqpodld, accessed on 6 June 2026.

The present study provides evidence supporting that persistent BoDV-1 infection is associated with altered *N*-glycan maturation of PrP in rat astroglial C6 cells, possibly involving reduced activity of the ETS1-MGAT5/GnTV pathway as well as reduced GnTV-dependent β1-6-branched complex *N*-glycan signals (Figure 10). These changes were accompanied by a shift of PrP toward lower-molecular-weight glycoform species and decreased SOD activity. These findings suggest *N*-glycan branching of PrP as a virus-sensitive regulatory checkpoint that may affect host glycosylation control in this C6 cell model, with potential implications for impaired astroglial antioxidative activity and neuropathogenesis induced by BoDV-1 infection.

## Figures and Tables

**Table 1:** Summary of fluorescent intensity in SAF83 flow-cytometric analysis.

Confluency	Corrected Geo Mean, C6	Corrected Geo Mean, C6/BoDV-1	C6/BoDV-1/C6 Ratio	Corrected Median, C6	Corrected Median, C6/BoDV-1	C6/BoDV-1/C6 Ratio
100%	336.31	320.67	0.95	369.93	359.96	0.97
50%	314.88	321.17	1.02	344.63	338.28	0.98
25%	354.34	351.89	0.99	351.38	348.32	0.99

Corrected values were calculated by subtracting the corresponding secondary-antibody-only negative-control value from the SAF83-stained value. Ratios were calculated as corrected C6/BoDV-1 value divided by corrected C6 value.

## Data Availability

The original contributions presented in this study are included in the article. Further inquiries can be directed to the corresponding author.

## Supplementary Materials

The following supporting information can be downloaded at https://www.mdpi.com/article/10.3390/microorganisms14081759/s1, Figure S1: Confirmation of BoDV-1 infection in C6/BoDV-1 cells by Western blotting; Figure S2: Confirmation of BoDV-1 infection in C6/BoDV-1 cells by RT-PCR.

## References

[1] Prusiner S.B. (1998). Prions. Proc. Natl. Acad. Sci. USA.

[2] Aguzzi A., Sigurdson C., Heikenwaelder M. (2008). Molecular mechanisms of prion pathogenesis. Annu. Rev. Pathol..

[3] Mead S., Hermann P., Mok T.H., Parchi P., Zerr I. (2026). Genetic causes and modifiers of prion diseases. Lancet Neurol..

[4] Pan K.M., Baldwin M., Nguyen J., Gasset M., Serban A., Groth D., Mehlhorn I., Huang Z., Fletterick R.J., Cohen F.E. (1993). Conversion of alpha-helices into beta-sheets features in the formation of the scrapie prion proteins. Proc. Natl. Acad. Sci. USA.

[5] Eghiaian F., Grosclaude J., Lesceu S., Debey P., Doublet B., Treguer E., Rezaei H., Knossow M. (2004). Insight into the PrP^C^-->PrP^Sc^ conversion from the structures of antibody-bound ovine prion scrapie-susceptibility variants. Proc. Natl. Acad. Sci. USA.

[6] Hara H., Sakaguchi S. (2020). N-terminal regions of prion protein: Functions and roles in prion diseases. Int. J. Mol. Sci..

[7] Wulf M.A., Senatore A., Aguzzi A. (2017). The biological function of the cellular prion protein: An update. BMC Biol..

[8] Castle A.R., Gill A.C. (2017). Physiological functions of the cellular prion protein. Front. Mol. Biosci..

[9] Westergard L., Christensen H.M., Harris D.A. (2007). The cellular prion protein (PrP(C)): Its physiological function and role in disease. Biochim. Biophys. Acta.

[10] Puig B., Altmeppen H., Glatzel M. (2014). The GPI-anchoring of PrP: Implications in sorting and pathogenesis. Prion.

[11] Brown D.R. (2005). Neurodegeneration and oxidative stress: Prion disease results from loss of antioxidant defence. Folia Neuropathol..

[12] Sakudo A., Ikuta K. (2009). Prion protein functions and dysfunction in prion diseases. Curr. Med. Chem..

[13] Marin-Moreno A., Espinosa J.C., Torres J.M. (2020). Transgenic mouse models for the study of prion diseases. Prog. Mol. Biol. Transl. Sci..

[14] Cha S., Kim M.Y. (2023). The role of cellular prion protein in immune system. BMB Rep..

[15] Lawson V.A., Collins S.J., Masters C.L., Hill A.F. (2005). Prion protein glycosylation. J. Neurochem..

[16] Hill A.F., Joiner S., Beck J.A., Campbell T.A., Dickinson A., Poulter M., Wadsworth J.D., Collinge J. (2006). Distinct glycoform ratios of protease resistant prion protein associated with *PRNP* point mutations. Brain.

[17] Priola S.A., Lawson V.A. (2001). Glycosylation influences cross-species formation of protease-resistant prion protein. EMBO J..

[18] Nishina K.A., Deleault N.R., Mahal S.P., Baskakov I., Luhrs T., Riek R., Supattapone S. (2006). The stoichiometry of host PrP^C^ glycoforms modulates the efficiency of PrP^Sc^ formation *in vitro*. Biochemistry.

[19] Katorcha E., Makarava N., Savtchenko R., Baskakov I.V. (2015). Sialylation of the prion protein glycans controls prion replication rate and glycoform ratio. Sci. Rep..

[20] Ermonval M., Mouillet-Richard S., Codogno P., Kellermann O., Botti J. (2003). Evolving views in prion glycosylation: Functional and pathological implications. Biochimie.

[21] Schilling K.M., Jorwal P., Ubilla-Rodriguez N.C., Assafa T.E., Gatdula J.R.P., Vultaggio J.S., Harris D.A., Millhauser G.L. (2023). *N*-glycosylation is a potent regulator of prion protein neurotoxicity. J. Biol. Chem..

[22] Beraldo F.H., Ostapchenko V.G., Xu J.Z., Di Guglielmo G.M., Fan J., Nicholls P.J., Caron M.G., Prado V.F., Prado M.A.M. (2018). Mechanisms of neuroprotection against ischemic insult by stress-inducible phosphoprotein-1/prion protein complex. J. Neurochem..

[23] Lopes M.H., Hajj G.N., Muras A.G., Mancini G.L., Castro R.M., Ribeiro K.C., Brentani R.R., Linden R., Martins V.R. (2005). Interaction of cellular prion and stress-inducible protein 1 promotes neuritogenesis and neuroprotection by distinct signaling pathways. J. Neurosci..

[24] Zanata S.M., Lopes M.H., Mercadante A.F., Hajj G.N., Chiarini L.B., Nomizo R., Freitas A.R., Cabral A.L., Lee K.S., Juliano M.A. (2002). Stress-inducible protein 1 is a cell surface ligand for cellular prion that triggers neuroprotection. EMBO J..

[25] Sakudo A., Lee D.C., Li S., Nakamura T., Matsumoto Y., Saeki K., Itohara S., Ikuta K., Onodera T. (2005). PrP cooperates with STI1 to regulate SOD activity in PrP-deficient neuronal cell line. Biochem. Biophys. Res. Commun..

[26] Sakudo A., Lee D.C., Saeki K., Nakamura Y., Inoue K., Matsumoto Y., Itohara S., Onodera T. (2003). Impairment of superoxide dismutase activation by N-terminally truncated prion protein (PrP) in PrP-deficient neuronal cell line. Biochem. Biophys. Res. Commun..

[27] Sakudo A., Lee D.C., Nishimura T., Li S., Tsuji S., Nakamura T., Matsumoto Y., Saeki K., Itohara S., Ikuta K. (2005). Octapeptide repeat region and N-terminal half of hydrophobic region of prion protein (PrP) mediate PrP-dependent activation of superoxide dismutase. Biochem Biophys. Res. Commun..

[28] de-Souza-Ferreira M., Ferreira E.E., de-Freitas-Junior J.C.M. (2023). Aberrant *N*-glycosylation in cancer: MGAT5 and beta1,6-GlcNAc branched *N*-glycans as critical regulators of tumor development and progression. Cell. Oncol..

[29] Ko J.H., Miyoshi E., Noda K., Ekuni A., Kang R., Ikeda Y., Taniguchi N. (1999). Regulation of the GnT-V promoter by transcription factor Ets-1 in various cancer cell lines. J. Biol. Chem..

[30] Kizuka Y., Taniguchi N. (2016). Enzymes for *N*-glycan branching and their genetic and nongenetic regulation in cancer. Biomolecules.

[31] Kizuka Y. (2024). Regulation of intracellular activity of *N*-glycan branching enzymes in mammals. J. Biol. Chem..

[32] Yale A.R., Kim E., Gutierrez B., Hanamoto J.N., Lav N.S., Nourse J.L., Salvatus M., Hunt R.F., Monuki E.S., Flanagan L.A. (2023). Regulation of neural stem cell differentiation and brain development by MGAT5-mediated *N*-glycosylation. Stem Cell Rep..

[33] Jungback N., Vollmuth Y., Mogele T., Grochowski P., Schlegel J., Schaller T., Markl B., Herden C., Matiasek K., Tappe D. (2025). Neuropathology, pathomechanism, and transmission in zoonotic Borna disease virus 1 infection: A systematic review. Lancet Infect. Dis..

[34] Reinmiedl J., Schulz H., Ruf V.C., Hernandez Petzsche M.R., Rissland J., Tappe D. (2022). Healthcare-associated exposure to Borna disease virus 1 (BoDV-1). J. Occup. Med. Toxicol..

[35] Kamitani W., Ono E., Yoshino S., Kobayashi T., Taharaguchi S., Lee B.J., Yamashita M., Kobayashi T., Okamoto M., Taniyama H. (2003). Glial expression of Borna disease virus phosphoprotein induces behavioral and neurological abnormalities in transgenic mice. Proc. Natl. Acad. Sci. USA.

[36] Honda T., Fujino K., Okuzaki D., Ohtaki N., Matsumoto Y., Horie M., Daito T., Itoh M., Tomonaga K. (2011). Upregulation of insulin-like growth factor binding protein 3 in astrocytes of transgenic mice that express Borna disease virus phosphoprotein. J. Virol..

[37] Teng D., Ueda K., Honda T. (2023). Impact of Borna disease virus infection on the transcriptome of differentiated neuronal cells and its modulation by antiviral treatment. Viruses.

[38] Marty F.H., Bettamin L., Thouard A., Bourgade K., Allart S., Larrieu G., Malnou C.E., Gonzalez-Dunia D., Suberbielle E. (2022). Borna disease virus docks on neuronal DNA double-strand breaks to replicate and dampens neuronal activity. iScience.

[39] Monnet C., Marthiens V., Enslen H., Frobert Y., Sobel A., Mege R.M. (2003). Heterogeneity and regulation of cellular prion protein glycoforms in neuronal cell lines. Eur. J. Neurosci..

[40] Vagin O., Tokhtaeva E., Yakubov I., Shevchenko E., Sachs G. (2008). Inverse correlation between the extent of *N*-glycan branching and intercellular adhesion in epithelia. Contribution of the Na,K-ATPase beta1 subunit. J. Biol. Chem..

[41] Bonnaud E.M., Szelechowski M., Betourne A., Foret C., Thouard A., Gonzalez-Dunia D., Malnou C.E. (2015). Borna disease virus phosphoprotein modulates epigenetic signaling in neurons to control viral replication. J. Virol..

[42] Jie J., Xu X., Xia J., Tu Z., Guo Y., Li C., Zhang X., Wang H., Song W., Xie P. (2018). Memory impairment induced by Borna disease virus 1 infection is associated with reduced H3K9 acetylation. Cell. Physiol. Biochem..

[43] Carbone K.M., Rubin S.A., Sierra-Honigmann A.M., Lederman H.M. (1993). Characterization of a glial cell line persistently infected with borna disease virus (BDV): Influence of neurotrophic factors on BDV protein and RNA expression. J. Virol..

[44] Benda P., Lightbody J., Sato G., Levine L., Sweet W. (1968). Differentiated rat glial cell strain in tissue culture. Science.

[45] Nakamura Y., Takahashi H., Shoya Y., Nakaya T., Watanabe M., Tomonaga K., Iwahashi K., Ameno K., Momiyama N., Taniyama H. (2000). Isolation of Borna disease virus from human brain tissue. J. Virol..

[46] Sakudo A., Tanaka Y., Ikuta K. (2011). Capture of infectious borna disease virus using anionic polymer-coated magnetic beads. Neurosci. Lett..

[47] Sakudo A., Onodera T., Ikuta K. (2008). PrP^Sc^ level and incubation time in a transgenic mouse model expressing Borna disease virus phosphoprotein after intracerebral prion infection. Neurosci. Lett..

[48] Sasaki S., Ogawa H., Katoh H., Honda T. (2024). Suppression of Borna disease virus replication during its persistent infection using the CRISPR/Cas13b system. Int. J. Mol. Sci..

[49] Taylor D.R., Hooper N.M. (2006). The prion protein and lipid rafts. Mol. Membr. Biol..

[50] Walmsley A.R., Zeng F., Hooper N.M. (2001). Membrane topology influences *N*-glycosylation of the prion protein. EMBO J..

[51] Lau K.S., Partridge E.A., Grigorian A., Silvescu C.I., Reinhold V.N., Demetriou M., Dennis J.W. (2007). Complex *N*-glycan number and degree of branching cooperate to regulate cell proliferation and differentiation. Cell.

[52] Dennis J.W., Lau K.S., Demetriou M., Nabi I.R. (2009). Adaptive regulation at the cell surface by *N*-glycosylation. Traffic.

[53] Huang R., Gao H., Zhang L., Jia J., Liu X., Zheng P., Ma L., Li W., Deng J., Wang X. (2012). Borna disease virus infection perturbs energy metabolites and amino acids in cultured human oligodendroglia cells. PLoS ONE.

[54] Brown D.R., Besinger A. (1998). Prion protein expression and superoxide dismutase activity. Biochem. J..

[55] Milhavet O., McMahon H.E., Rachidi W., Nishida N., Katamine S., Mange A., Arlotto M., Casanova D., Riondel J., Favier A. (2000). Prion infection impairs the cellular response to oxidative stress. Proc. Natl. Acad. Sci. USA.

[56] Spiers J.G., Chen H.C., Steinert J.R. (2023). Redox mechanisms and their pathological role in prion diseases: The road to ruin. PLoS Pathog..

[57] Day R.M., Suzuki Y.J. (2006). Cell proliferation, reactive oxygen and cellular glutathione. Dose Response.

[58] Bae Y.S., Oh H., Rhee S.G., Yoo Y.D. (2011). Regulation of reactive oxygen species generation in cell signaling. Mol. Cells.

