# Supplementary material for: BoDV-1 Infection Is Associated with Altered Prion Protein Glycoform Maturation and Reduced ETS1-MGAT5/GnTV Pathway Activity in Rat C6 Astroglial Cells
Source: Microorganisms. 2026 Aug 10;14(8):1759. doi: 10.3390/microorganisms14081759 (PMC13515920; doi:10.3390/microorganisms14081759)
Supplement: Supplementary file 1 [file microorganisms-14-01759-s001.zip › microorganisms-4449326-supplementary.pdf]

Supplementary Materials

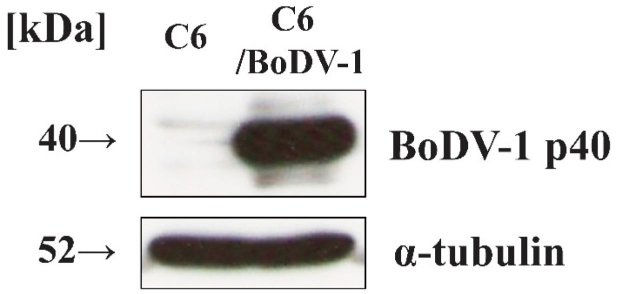

Supplementary Figure S1. Confirmation of BoDV-1 infection in C6/BoDV-1 cells by Western blotting. C6 and C6/BoDV-1 cells were cultured at 100% confluency and subjected to Western blotting with anti-BoDV-1 p40 and anti-α-tubulin, as described in Materials and Methods. α-tubulin was detected on a separate membrane loaded with the same samples (20 μg protein/lane). The molecular masses (kDa) are shown on the left-hand side of each blot. Data from one representative experiment are shown.

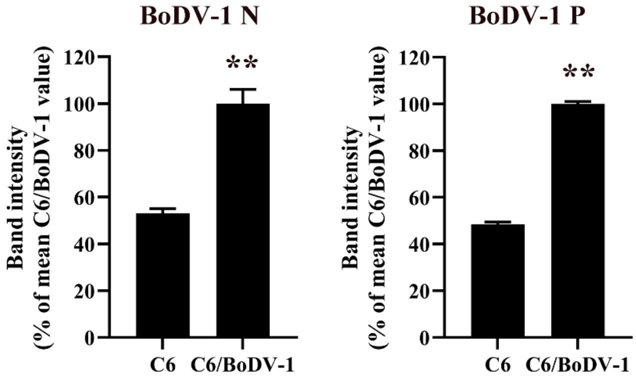

Supplementary Figure S2. Confirmation of BoDV-1 infection in C6/BoDV-1 cells by RT-PCR. Total RNA was extracted from uninfected C6 cells and BoDV-1-infected C6 cells (C6/BoDV-1) harvested at 100% confluency on three independent days. These samples are referred to as three independently prepared RNA samples ($n = 3$). The RNA samples were reverse-transcribed, and PCR was used to amplify BoDV-1 N and P gene fragments. PCR band intensities were quantified using ImageJ. For each viral gene, the mean band intensity of the three C6/BoDV-1 samples was defined as 100%, and individual values from both C6 and C6/BoDV-1 samples were expressed as percentages of this mean reference value. Thus, the 100% value represents the mean of the C6/BoDV-1 reference group, whereas the error bars indicate the variability among the three independently prepared RNA samples. Data are shown as mean ± SEM. $^{**}p < 0.01$, significant difference compared with the uninfected counterpart.
